# When Prior Exposure Doesn’t Protect: Imported Plasmodium falciparum Malaria in a Returning Traveler With Waning Immunity

**DOI:** 10.7759/cureus.114360

**Published:** 2026-08-11

**Authors:** My Nguyen, Nabia Khan, Neeja Patel, Adela Greeley, Christopher Moriates

**Affiliations:** 1 David Geffen School of Medicine, University of California, Los Angeles, Los Angeles, USA; 2 Department of Anesthesiology, University of California, Los Angeles, Los Angeles, USA; 3 Department of Medicine, University of California, Los Angeles, Los Angeles, USA; 4 Department of Medicine, Greater Los Angeles Veterans Affairs Healthcare System, Los Angeles, USA

**Keywords:** anti-malarial medications, atovaquone, glucose-6-phosphate dehydrogenase deficiency, imported malaria, oxidative hemolysis, plasmodium falciparum, visiting friends and relatives travelers

## Abstract

Malaria remains a leading cause of infectious morbidity worldwide, yet imported cases present a diagnostic challenge in non-endemic settings. We report a case of imported malaria in a previously healthy 35-year-old man, who presented with increasing fatigue, jaundice, and cyclical fevers following travel to an endemic area. Peripheral blood smear demonstrated *Plasmodium falciparum* trophozoites, prompting initiation of antimalarial therapy. Further testing detected previously undiagnosed glucose-6-phosphate dehydrogenase (G6PD) deficiency. The patient improved clinically and was discharged after five days of hospitalization.

This case illustrates two important considerations in the management of imported malaria. First, Visiting Friends and Relatives (VFR) travelers, those who grew up in malaria-endemic areas and now reside in other countries, represent the highest-risk group for imported malaria. Nonetheless, this population is among the least likely to use chemoprophylaxis - a pattern driven by a misconception of residual immunity shared by both patients and clinicians. As a result, this misconception can delay diagnosis: the patient’s presumed immunity contributed to an initial misattribution of symptoms to a viral illness, highlighting the need for a low threshold to test for malaria in febrile returning travelers. Second, this case underscores the importance of G6PD testing before initiating antimalarial regimens, as many first-line medications carry the risk of oxidative hemolysis in patients with G6PD deficiency. Recognition of these issues is imperative for safe and effective management of malaria in non-endemic settings.

## Introduction

Malaria is a parasitic disease caused by protozoa of the genus *Plasmodium*, transmitted mainly through the bite of an infected female Anopheles mosquito [1]. The five species include *Plasmodium* *falciparum​​​​​​​* (*P. falciparum*), *P. vivax*, *P. malariae*, *P. ovale*, and *P. knowlesi*, with *P. falciparum* bearing the greatest global burden of severe disease and death [2]. Symptoms range from cyclical fever, fatigue, and headache to life-threatening complications, including severe anemia, coma, convulsions, acute respiratory distress syndrome, and multi-organ failure [2]. Cerebral malaria, most commonly caused by *P. falciparum*, typically presents as seizures and altered mental status, carrying the risk of permanent neurological damage [2].

Incubation periods differ by species; *P. falciparum* infection typically presents within two weeks of infection, though asymptomatic parasitemia can persist for months to years in partially immune individuals [2]. Groups with increased risk include young children, pregnant women, immunocompromised individuals, and travelers to endemic regions [2]. Among those at increased risk of contracting malaria, studies have shown that Visiting Friends and Relatives (VFR) travelers, those who grew up in malaria-endemic areas and now reside in other countries, are disproportionately affected due to lower rates of prophylaxis use and underestimated susceptibility [3]. Partial immunity to malaria is acquired through continual exposure to *Plasmodium *in endemic areas and is maintained only through ongoing re-exposure, as immunity is lost shortly after leaving the region [4]. This decline occurs for several reasons. Firstly, protective cytokine responses, including IFN-gamma, TNF-alpha, and IL-10, decline significantly within as little as 6 months of cessation of exposure [4]. Additionally, antibody responses to malaria antigens depend on the antigen itself, with IgG responses to infected erythrocyte surface antigens being lower than those to other antigens [5]. Thus, prior residence in an endemic area confers less protection than is often assumed.

Peripheral blood smear remains the gold standard diagnostic test for malaria, enabling identification of parasitic forms such as trophozoites, schizonts, and gametocytes [2]. Management depends on parasite species, local resistance patterns, and comorbidities such as G6PD deficiency. Artemisinin-based combination therapies (ACTs) are preferred for uncomplicated P. falciparum, providing rapid efficacy and mitigating resistance. Alternatives such as atovaquone-proguanil are used where resistance is a concern or if the patient is of childbearing age [2]. For *P. vivax* and *P. ovale*, liver-stage eradication requires agents such as primaquine or tafenoquine; however, these medications increase the risk of hemolysis in individuals with G6PD deficiency, so screening is required prior to administration [2,6]. The hemolysis sequence begins when an oxidant drug is administered, leading to the formation of metabolites that then trigger the release of hemoglobin in red blood cells and the spleen. This process sets off a downstream cascade that can present in affected individuals as severe anemia, jaundice, or gallstones and may lead to conditions such as renal damage, thromboembolic risk, acute inflammation, and, in severe cases, death [6]. Other drugs that carry a risk of hemolysis include dapsone, methylene blue, nitrofurantoin, phenazopyridine, primaquine, rasburicase, and toluidine blue [6]. Clinical decision-making must integrate local guidelines, resistance data, and patient-specific risks to optimize outcomes.

## Case presentation

A man in his thirties with no significant medical history presented to the emergency room in Los Angeles with two syncopal episodes and worsening fatigue, jaundice, and fevers. He traveled to Morocco for five weeks and then to Nigeria for four weeks, returning to the United States two weeks prior to his presentation. He did not use malaria prophylaxis, believing it unnecessary since he was born in Nigeria. After childhood in Nigeria, he lived in the United Kingdom before moving to the United States in 2007. He works as a self-employed engineer and travels to Dubai twice yearly for work. Two weeks after returning from Morocco and Nigeria, the patient noticed severe fatigue and fevers. He first visited his primary care doctor, who reassured him that it was likely a viral illness and discussed supportive care. As symptoms persisted, the patient took unprescribed levofloxacin without improvement. He had multiple episodes of diarrhea in the past week and felt diffuse chills. Before admission, he had sudden dizziness and had fallen without loss of consciousness or head trauma. He described major activity limitations-unable to walk more than 30 feet without breathlessness-as well as a 15-pound weight loss and poor appetite since symptoms began.

The patient reported that he was a social drinker with no cigarette use or recreational drug use. He was currently sexually active with one female partner.

At the time of admission, the patient had recorded fevers with a maximum temperature of 101.8 °F. The physical exam was notable for a fatigued appearance, dry mucous membranes, and scleral jaundice, without splenomegaly, hepatomegaly, or pallor. His heart sounds were normal, and his lungs were clear to auscultation without wheezing or crackles.

Initial laboratory studies revealed leukocytosis, thrombocytopenia, and hyponatremia (Table 1). Hemolytic markers, including elevated lactate dehydrogenase (LDH; 385 U/L), reticulocytosis (5.3% reticulocytes), and a low haptoglobin level, were present. Diagnostic imaging via chest X-ray showed no acute cardiopulmonary process. Blood cultures were obtained, and urinalysis was unremarkable. In addition, a broad infectious work-up, including a respiratory viral panel and testing for HIV, coccidioidomycosis, tuberculosis, and rapid plasma reagin (RPR), was sent at the time of admission, and ultimately returned negative.

**Table 1:** Lab studies from time of admission to the hospital ALT: alanine aminotransferase; AST: aspartate aminotransferase; BUN: blood urea nitrogen; G6PD, glucose-6-phosphate dehydrogenase; Hgb: hemoglobin; LDH: lactate dehydrogenase; RBC: red blood cell; WBC: white blood cell

Test	Value	Reference range
WBC	12,650 cells/microliter	4500-11,000 cells/microliter
Metamyelocytes	5.9%	0%
% Parasitemia	0.34%	0%
Hgb	8 g/dL	Male: 13.5- 17.5 g/dL, Female: 12.0-16.0 g/dL
Hematocrit	24.2%	Male: 41%-53%, Female: 36%-48%
Platelet count	115,000 cells/microliter	150,000-400,000 cells/microliter
RBC	2.84 million cells/microliter	Male: 4.7 to 6.1 million cells/microliter, Female: 4.2 to 5.4 million cells/microlitercL
Reticulocyte %	5.3%	0.5%-1.5%
Serum sodium	131 mEq/L	136-146 mEq/L
BUN	20 mg/dL	7-18 mg/dL
Creatinine	1.2 mg/dL	0.6-1.2 mg/dL
Albumin	2.5 g/dL	3.5-5.5 g/dL
AST	78 U/L	12-38 U/L
ALT	96 U/L	10-40 U/L
LDH	385 U/L	45-200 U/L
Haptoglobin	<10	50-220 mg/dL
Total bilirubin	1.3 mg/dL	0.1-1.0 mg/dL
G6PD level	2.2 U/g Hb	5-15 U/g Hb

**Figure 1:**
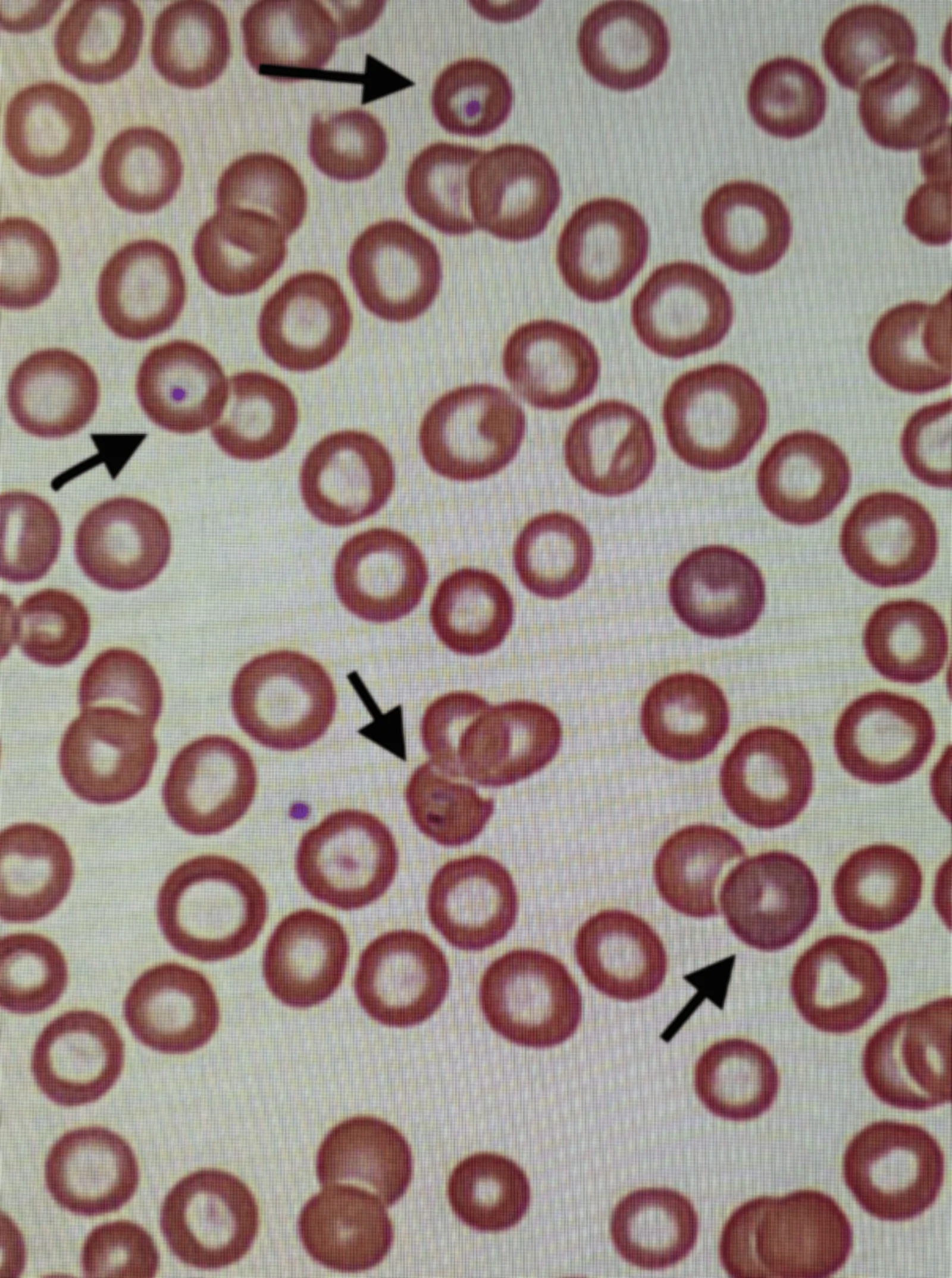
Screenshot of peripheral blood smear taken at time of admission, revealing P. falciparum trophozoites within infected red blood cells

The travel history was the critical pivot point, leading the medical team to obtain and personally review a stat peripheral blood smear at the time of admission. The smear revealed immature “ring forms” characteristic of intraerythrocytic *P. falciparum* trophozoites (Figure 1), raising immediate concern for malaria in the context of the patient's recent travel. Subsequently, a G6PD panel revealed a previously undiagnosed G6PD deficiency.

Treatment

Upon recognition of the patient's recent travel history, lack of malaria prophylaxis, and strong suspicion of a parasitic infection, a single empiric dose of atovaquone-proguanil was administered in the emergency department while peripheral smear results were pending. Following definitive identification of *P. falciparum* malaria infection, a full treatment course of atovaquone-proguanil (four tabs daily for three days total duration) was prescribed. Given the confirmed G6PD deficiency, primaquine and tafenoquine were withheld. As P. falciparum does not form liver-stage hypnozoites, no additional therapy was required. The hyponatremia, which was managed conservatively with fluid restriction, was thought to most likely be attributed to malaria-causing inappropriate antidiuretic hormone secretion.

Outcome and follow-up

The patient demonstrated rapid clinical improvement following initiation of atovaquone-proguanil. He was discharged after five days of inpatient care without complications. Throughout the hospital course, he did not develop features of severe malaria, including neurologic sequelae, respiratory failure, or renal impairment. The Department of Health was notified in accordance with surveillance requirements for confirmed malaria cases.

## Discussion

This case illustrates several clinically important principles in the evaluation and management of imported malaria in non-endemic settings.

Firstly, VFR travelers to malaria-endemic regions encounter an increased, often unrecognized, risk due to both patients' and clinicians’ misconceptions [7]. As protective immunity wanes after relocation, VFR travelers can develop severe malaria, similar to non-immune individuals. A Swedish study found that African immigrants who had lived in a non-endemic country for more than 15 years had an equivalent risk of severe malaria to that of non-immune travelers [8]. Nonetheless, many VFR travelers mistakenly believe that prior residence offers immunity against parasitic infections, even with these risks, and choose not to take chemoprophylaxis before traveling [7]. Social pressure may further discourage the use of prophylaxis. Many of the VFR travelers reported that if they got prophylaxis, they would feel as though they had “rejected” their community [9]. Unsurprisingly, among American residents who developed malaria, 95% did not use or did not comply with the prophylaxis regimen CDC had recommended [10]. Clinicians can play a prevention role by actively screening patients from endemic regions about travel plans and by addressing the misconception that childhood exposure to infections confers lasting protection.

Secondly, failing to recognize the increased risk for VFR travelers and to consider recent travel history likely led to a delayed diagnosis in this case, as the patient’s primary care provider initially attributed the symptoms to presumed viral illness. Globally, 13% of returning travelers with malaria were misdiagnosed at initial presentation and discharged with over-the-counter pain killers, only for them to return to the emergency department with worsening symptoms [2]. This can be attributed to nonspecific symptoms that overlap considerably with viral upper respiratory infections and gastroenteritis, making it difficult to distinguish without clear clinical context. Notably, cough occurs in approximately 36% of *P. falciparum* malaria cases and up to 53% of *P. vivax/ovale* malaria cases, and may be accompanied by tachypnea and impaired gas transfer, features that can readily be mistaken for pneumonia [11]. Laboratory findings such as thrombocytopenia, elevated LDH, and anemia may provide useful diagnostic clues, but travel history remains the critical trigger to consider malaria in the differential diagnosis. To address the ambiguous presentation and nonspecific symptoms of malaria, clinicians should have a low threshold to inquire about travel history and to test for malaria, including obtaining a peripheral blood smear, in febrile patients, even if the patient considers themselves “immune”.

Lastly, G6PD testing is essential before administering primaquine or tafenoquine, as these agents may precipitate severe oxidative hemolysis in G6PD-deficient patients. It is especially relevant since G6PD deficiency and malaria share a similar geographical distribution. G6PD affects an estimated 400 million people worldwide, with the majority from Africa, Southeast Asia, the Middle East, and the Mediterranean [12]. Thus, VFR travelers returning from endemic areas, the population of focus in this case presentation, are disproportionately likely to carry G6PD deficiency, making testing essential. In this case, the patient's G6PD deficiency was entirely asymptomatic until diagnosis; had *P. vivax* or *P. ovale* malaria been identified, initiation of primaquine without prior testing could have resulted in life-threatening hemolytic anemia. An interesting consideration is whether the patient's G6PD deficiency may have conferred partial protection against cerebral malaria - a phenomenon suggested by genome-wide association studies [13] - while possibly increasing susceptibility to severe malarial anemia.

## Conclusions

*P. falciparum* malaria is a medical emergency demanding prompt diagnosis and treatment. Both physicians and patients may mistakenly believe that those previously from endemic regions will have retained immunity, but this case underscores that these VFR travelers who now reside in other countries represent the highest-risk group for imported malaria. The case highlights the value of obtaining a thorough travel history, maintaining a high level of suspicion for malaria in returning travelers, and testing for G6PD deficiency to guide appropriate antimalarial medication administration.
